# Diseases of the Stomach

**Published:** 1875-04

**Authors:** 


*The Diseases of the Stomach.* Being the Third Edition of the Diagnosis and Treatment of the Varieties of Dyspepsia. Revised and Enlarged. By Wilson Fox, M. D., F. R. C. P., F. R. S. Philadelphia: Henry C. Lea, 1875. Buffalo: T. Butler & Son.

Since the publication of the two former editions of the work on Dyspepsia the author has enlarged his plan by the addition of articles on Ulcer and Cancer of the Stomach, and a consideration of various symptoms by which derangements of the stomach were revealed. Under the present form it may, therefore, be considered as a special work upon diseases of the stomach; as such it will be found to be possessed of considerable value.

The contents are divided into two parts, first: Symptomatology of the Stomach; second: Special Diseases. Under the first section are considered the appearance of the tongue, derangement of appetite, thirst, flatulence, vomiting and indigestion. Under Special Diseases the author groups atonic dyspepsia, neuroses, acute and chronic catarrh, ulcer, cancer, hemorrhage, hypertrophy, stricture of the cardiac orifice, obstruction of the pylorus, dilitation, softening, rupture and tubercle.

From the foregoing it will be seen that the work is quite comprehensive. The various topics embraced are all well considered, and as a book of reference or for study it will be found to be of much value.

